# Stereotaxical Infusion of Rotenone: A Reliable Rodent Model for Parkinson's Disease

**DOI:** 10.1371/journal.pone.0007878

**Published:** 2009-11-18

**Authors:** Nian Xiong, Jinsha Huang, Zhentao Zhang, Zhaowen Zhang, Jing Xiong, Xingyuan Liu, Min Jia, Fang Wang, Chunnuan Chen, Xuebing Cao, Zhihou Liang, Shenggang Sun, Zhicheng Lin, Tao Wang

**Affiliations:** 1 Department of Neurology, Union Hospital, Tongji Medical College, Huazhong University of Science and Technology, Hubei, China; 2 Department of Psychiatry, Harvard Medical School, Boston, Massachusetts, United States of America; 3 Mailman Research Center, McLean Hospital, Belmont, Massachusetts, United States of America; University of Nebraska, United States of America

## Abstract

A clinically-related animal model of Parkinson's disease (PD) may enable the elucidation of the etiology of the disease and assist the development of medications. However, none of the current neurotoxin-based models recapitulates the main clinical features of the disease or the pathological hallmarks, such as dopamine (DA) neuron specificity of degeneration and Lewy body formation, which limits the use of these models in PD research. To overcome these limitations, we developed a rat model by stereotaxically (ST) infusing small doses of the mitochondrial complex-I inhibitor, rotenone, into two brain sites: the right ventral tegmental area and the substantia nigra. Four weeks after ST rotenone administration, tyrosine hydroxylase (TH) immunoreactivity in the infusion side decreased by 43.7%, in contrast to a 75.8% decrease observed in rats treated systemically with rotenone (SYS). The rotenone infusion also reduced the DA content, the glutathione and superoxide dismutase activities, and induced alpha-synuclein expression, when compared to the contralateral side. This ST model displays neither peripheral toxicity or mortality and has a high success rate. This rotenone-based ST model thus recapitulates the slow and specific loss of DA neurons and better mimics the clinical features of idiopathic PD, representing a reliable and more clinically-related model for PD research.

## Introduction

PD is a common, neurodegenerative disorder characterized by tremor at rest, rigidity, bradykinesia, hypokinesia, and postural instability. Its pathological hallmarks include the loss of DA neurons of the substantia nigra-striatum system and the presence of proteinacious cytoplasmic inclusions, known as Lewy bodies, in the remaining DA neurons[1]. Although PD has been studied intensively for almost two centuries, since James Parkinson gave the first detailed description of the disease in 1817[2], the etiology and pathogenesis of the disease remain unknown.

A well-established and clinically-related model is needed urgently to enable the study of PD pathogenesis and to facilitate the search for effective treatments for PD. PC12[3], [4], [5], SH-SY5Y[6], [7] cells, and primary dissociated midbrain DA cells[8] have been extensively used as PD cell models. Because the microenvironment of *in vitro* cultured cells is substantially different from that of the living brain tissues, the value of cell models is limited. Therefore, increasing attention has been paid to animal models of PD. So far, there are two kinds of animal models of PD: traditional neurotoxin models and genetic mouse models. The genetic models of PD, established by inducing mutations associated with familial forms of PD, are of limited value because most of the murine genetic models do not display the characteristic loss of DA neurons observed in PD, as they are still at an early stage of this disease. Moreover, it has been reported that, collectively, these genetic mutations are found in less than 10% of PD patients in the US[9].

The two most popular neurotoxin-based models of PD are generated by the administration of 6-hydroxydopamine (6-OHDA) and 1-methyl-4-phenyl-1,2,3,6-tetrahydropyridine (MPTP). The intracerebral infusion of 6-OHDA in mice, rats, cats, and nonhuman primates produces a rapid loss of dopaminergic terminals and dopaminergic neurons, which results in an anterograde degeneration of the entire nigrostriatal dopaminergic system [10], [11], [12], [13]. However, alpha-synuclein expression is not found in these models [14]. As mitochondrial complex I deficiency was found to be a major factor responsible for neurodegeneration[15], and since Parkinson-like symptoms were found in drug (one of the main components is MPTP) addicts[16], MPTP, a potent inhibitor of complex I, has extensively been used to induce Parkinsonian symptoms in animals (mice, cats, and primates). However, these models do not have the chronic progression and extensive pathological involvement (such as serotoninergic and norepinephrinergic pathways) seen in PD [17], [18].

Like MPTP, rotenone is another complex I inhibitor and has been reported to possess highly selective toxicity on DA neurons *in vitro* [5], [19] and *in vivo* [15], [20]. The main differences between MPTP and rotenone are that rotenone facilitates the formation of alpha-synuclein fibrils[15] and easily crosses the blood brain barrier, as well as the cell membrane, because of its lipophilic nature; MPTP requires enzymatic conversion into MPP^+^ whose entry into neurons needs a dopamine transporter. Among the various animal models of PD, the rotenone model has recently drawn particular attention for two reasons: 1) it reproduces most of the motor symptoms and the histopathological features of PD, including Lewy bodies[21], [22], and 2) rotenone and other pesticides are powerful inhibitors of mitochondrial respiration, and recent epidemiological studies suggest an association of these toxic compounds with the higher incidence of sporadic Parkinsonism among the population of rural areas[23], [24].

Nevertheless, systemic administration of rotenone can cause peripheral toxicity, and the behavioral changes of animals might be the result of general health problems rather than a specific motor deficit characteristic of PD symptoms[25]. Recently, several researchers intracerebrally infused rotenone into the medial forebrain bundle (MFB) [26], [27] or into the substantia nigra pars compacta (SNc) [28] and reproduced neurochemical and neuropathological features of hemiparkinsonism in rats, but these models failed to reproduce the extensive pathological involvement seen in PD. Moreover, alpha-synuclein expression in the DA neurons was not mentioned in these studies [28], [29].

It is widely acknowledged that it is not only the substantia nigra and striatum, but also other regions of brain that are responsible for PD [17]. Among these brain regions, the ventral tegmental area (VTA), which contains abundant DA neurons, is remarkably important for the pathogenesis of PD [30], [31]. It has even been reported that VTA lesions are responsible for the hypokinesia of PD [32]. In this study, rotenone was directly infused stereotaxically into the right VTA and SNc of rats. Four weeks after the infusion, the behavioral profiles, biogenic amine levels in the striatum, oxidative stress levels, and TH immunoreactivities were assessed. More importantly, compared to the SYS model, the ST model was further validated by the detection of alpha-synuclein expression and the ultrastructural changes in SNc.

## Materials and Methods

### Animals

The experiments described in this paper were approved by the Ethical Committee on Animal Experimentation of Tongji Medical College, Huazhong University of Science and Technology, China. Inbred adult female Sprague-Dawley rats (220–260 g, from the Center of Experimental Animals, Tongji Medical College, Huazhong University of Science and Technology, China) were used. The animals were maintained under standard conditions with 12-hour light/dark cycles and 22±2°C and 60±5% humidity. They had access to standard laboratory chow and acidified water *ad libitum*. Rats were randomly assigned to five groups: ST infusion of rotenone at 3 µg in 1 µl DMSO (3-µg group, n = 40), 6 µg in 1 µl DMSO (6-µg group, n = 15), 12 µg in 1 µl DMSO (12-µg group, n = 15), ST infusion of 1 µl DMSO (vehicle group) (n = 15), or SYS infusion of rotenone at 2 mg/kg/day (n = 12). The total period of the study was six months, and no accidental animal deaths took place during the study. For dose-response studies of the DA and 5-HT contents and the oxidative stress level in the ST model, animals from the 3-, 6- and 12-µg groups were sacrificed on the fourth week following the ST infusion of rotenone. For the time-response studies, animals from the 3-µg group were sacrificed on the second, fourth, sixth, and eighth weeks after surgery.

### Drugs and Chemicals

The rotenone, apomorphine (APO), DA, serotonin (5-HT), chloral hydrate, 3,3′-diaminobenzidine, hematoxylin, and Hoechst 33258 were purchased from Sigma (St. Louis, MO); Triton-X100, paraformaldehyde, dimethyl sulfoxide (DMSO), and EDTA were from Amresco (Solon, OH); rabbit anti-tyrosine hydroxylase polyclonal antibody and mouse anti-α-synuclein antibody were obtained from Santa Cruz (Santa Cruz, CA); biotinylated goat-anti-rabbit IgG, peroxidase-conjugated streptavidin, superoxide dismutase (SOD) activity colorimetric assay kit, and glutathione (GSH) colorimetric detection kit were from Abcam (Cambridge, MA); Cy3-conjugated goat-anti-rabbit IgG and FITC-conjugated donkey-anti-mouse IgG were purchased from Proteintech (Chicago, IL); and the malondialdehyde (MDA) colorimetric Assay Kit was from Oxis International (Beverly Hills, CA, USA). Sodium dodecyl sulfate (SDS) and other reagents were of analytical grade and procured locally. For the high performance liquid chromatography (HPLC) experiments, double-distilled water was filtered and deionized by using a Milli-Q system (Waters, Milford, MA).

### Rotenone Infusion

For ST infusion, the animals were anesthetized with chloral hydrate (400 mg/kg in 0.9% NaCl, I.P.) and fastened on a cotton bed over a stereotaxic frame (RWD Life Science, Shenzhen, China). Rotenone dissolved in DMSO was infused into the right VTA (AP: 5.0 mm; L: 1.0 mm; DV: 7.8 mm) at a flow rate of 0.2 µl/min. The needle was left in place for additional five minutes for complete diffusion of the drug. Rotenone was infused into the right SNc (AP: 5.0 mm; L: 2.0 mm; DV: 8.0 mm) at a flow rate of 0.2 µl/min, with a five-minute needle retention. After needle withdrawal, proper postoperative care was given until the animals recovered completely. The animals were given ibuprofen and penicillin in their drinking water for 24 hours to alleviate potential postsurgical discomfort and to prevent infection. For the SYS infusion, rotenone dissolved in sunflower oil was injected subcutaneously (2 mg/kg/d) into the back of these rats daily for four weeks.

### Spontaneous Rotation Behavior

Rats receiving ST infusion were intensively observed for any abnormal activity during the first 72 hours. One of the striking features following recovery from anesthesia was spontaneous circling. The animals were kept in a transparent cage following surgery, and the spontaneous rotations in the cage were counted over a period of one hour.

### APO-Induced Rotations

ST infusion rats were evaluated for APO-induced rotations from the second week after rotenone administration and tested every week in the first month, every two weeks in the next two months, and then every month for a total of six months. The rats were placed on a table (1 m×1 m) with a railing, allowed to acclimate to the environment for 10 minutes, and then administered an intraperitoneal injection of APO (1.5 mg/kg). Subsequent rotational behavior was then recorded for 30 minutes. Following the rotational testing, the animals were put back into their cages.

### DA and 5-HT Determination

The left and right striata were micro-punched from the brains, and the tissues were weighed wet. The tissues were immediately frozen and stored at −80°C until sonication in ice-cold 0.01 M HClO_4_ solution containing 0.01% EDTA [33], [34]. The supernatant (12,000 rpm for 10 minutes) was collected and injected (20 µl) into an HPLC system equipped with a fluorescence detector (Waters, MA). The Lichrosorb Column (C18, 10 µm, 25 cm×4.6 mm, Waters, MA) was employed, and the mobile phase consisted of trisodium citrate (0.02 M), sodium dihydrogen phosphate (0.05 M), methanol (40%), EDTA (0.028 g/L), and SDS (0.15 g/L). The solution was adjusted to pH 3.0 with 98% H_2_SO_4_, filtered through a 0.45-µm membrane, and degassed. The flow rate was set to 1.0 ml/min, and the column temperature was set at 40°C. After separation, DA and 5-HT were detected at the excitation wavelength of 280 nm and an emission wavelength of 315 nm.

### GSH and SOD Activity and MDA Level Determination

Animals were sacrificed, and the brains were washed with ice-cold 0.01 M sterilized phosphate buffered solution (PBS). The left and right midbrains were micro-punched and cut into small pieces and placed into glass bottles. The tissue pieces were then homogenized in ice-cold PBS by mechanical trituration. The homogenate was centrifuged at 3000 rpm for 10 minutes, and a supernatant was obtained, which was used for the determination of the GSH and SOD activity. All procedures were performed at 4°C and icepacks were used to maintain the temperature during the homogenization. The spectrophotometric assay for GSH involved oxidation of GSH by the sulfhydryl reagent 5,5′-dithio-bis(2-nitrobenzoic acid) to form the yellow derivative 5′-thio-2-nitrobenzoic acid, measurable at 412 nm[35]. The principle of the SOD activity determination is based on the inhibition of nitroblue tetrasolium reduction by the xanthine-xanthine oxidase system serving as a superoxide radical generator[36]. The SOD activity was expressed as units per mg tissue protein (U/mg protein). The MDA levels were analyzed using a method based on the reaction with thiobarbituric acid at 90-100°C. In the thiobarbituric acid test, MDA or MDA-like substances and thiobarbituric acid were mixed to allow reaction to produce a pink pigment with a maximum absorption at λ = 532 nm. The protein concentration was determined by the bicinchoninic acid method.

### Immunohistostaining

Coronal sections (5-µm) were cut through the striatum (at 1.2 mm caudal to the bregma) and the SNc (from −4.5 to −6.2 mm caudal to the bregma) by using a sledge microtome (Wetzlar, Germany). The sections were de-waxed, hydrated, and the endogenous peroxidase was quenched with 0.3% H_2_O_2_ for 30 minutes. After antigen retrieval, the slides were treated with 0.5% Triton-X100 for 30 minutes and 5% bovine serum albumin (BSA) for 30 minutes. The sections were first incubated with antibody/PBS (TH antibody, 1∶100 dilution) for 48 hours at 4°C and then incubated with the secondary biotinylated goat anti-rabbit IgG for 60 minutes and with peroxidase-conjugated streptavidin for 45 minutes. The immunoreactions were visualized by 3,3′-diaminobenzidine for 15-20 minutes. The slides from the anterior to posterior regions of the left and right striata were densitometrically observed and analyzed by employing the Image-Pro plus 6.0 software package. A design-based, unbiased, stereological method and a morphometry/image analysis system has been previously described for counting TH-positive cells[37]. In each section, the region of interest was outlined (SNc and VTA), and the TH-positive cells/mm^2^ in that region were selected and semiautomatically counted. For the double immunohistostaining, the sections were incubated with both antibodies/PBS (TH antibody, 1∶100 dilution and alpha-synuclein, 1∶100 dilution) for 48 hours at 4°C and then with Cy3-conjugated goat-anti-rabbit IgG (1∶100) and FITC-conjugated donkey-anti-mouse IgG (1∶100) diluted by 10 µg/ml Hoechst 33258 (the secondary antibody). The slides were then observed under a confocal fluorescence microscope and the pictures were still analyzed by Image-Pro plus 6.0.

### Ultrastructure of the SNc

Rats were deeply anesthetized on the first, second, third, and fourth week after operation and perfused through the aorta with ice-cold PBS and then with paraformaldehyde (4% wt/vol) and glutaraldehyde (1% wt/vol) in PBS[38]. The brains were post-fixed in 2.5% glutaraldehyde at 4°C for 6 hours after the separation. A 1-mm^3^ tissue block from the left and right SNc regions (−4.5 to −6.2 mm caudal to the bregma) was micro-punched, fixed in PBS containing 2.5% glutaraldehyde, and preserved at 4°C for further processing. The fragments were post-fixed in 1% osmium tetroxide in the same buffer, dehydrated in graded alcohols, embedded in Epon 812, sectioned with an ultramicrotome, and stained with uranyl acetate and lead citrate. The sections were then examined under a transmission electron microscope (TEM; Technai 10, Philips, Netherlands).

### Pathology of the Peripheral Organs

The pathological evaluation of the peripheral organs was performed on eight rats from each group. Four weeks after the rotenone treatment, the animals were deeply anesthetized and perfused through the aorta with ice-cold PBS and then with paraformaldehyde (4% wt/vol) in PBS. The heart, kidney, liver, lung, spleen, and stomach were taken out and immersed in 4% paraformaldehyde in PBS at 4°C for 24 hours before paraffin-embedding. The tissue sections were cut into 5-µm pieces, stained with hematoxylin and eosin (HE), and examined under a light microscope.

### Statistical Analyses

The statistical analyses were carried out using SPSS version 12.0 for Windows (SPSS, Chicago, IL, USA). Because all groups showed a normal distribution, the intergroup differences were assessed using parametric statistical methods, paired independent sample *t*-tests and a one-way analysis of variance (ANOVA). The results are presented as the means±SD. The *P* value considered statistically significant was *P*<0.05.

## Results

### Variability and Mortality

All the ST model rats showed typical behavioral features of PD, such as back hunching and stiffness, face-washing behaviors, bradykinesia, or hypokinesia. Ten of the twelve SYS models developed characteristic behavioral features of PD after rotenone infusion for four weeks. The remaining two rats developed the behavioral features after another four-week SYS infusion of rotenone. No animals died accidentally throughout the study.

### Spontaneous Rotation Behavior after Recovery from Anesthesia

After recovery from anesthesia, spontaneous rotations to the opposite side of the infusion site were observed in all the rats receiving infusions of rotenone into the VTA and SNc. The frequency of spontaneous rotations was 224±52 times per hour during the first day after surgery. This behavior showed no significant difference between the dose-response groups. The intensity of the behavior persisted for about 24 hours, gradually declined thereafter, and vanished after 48–72 hours.

### APO-Induced Rotations Advanced Gradually

**Figure 1:**
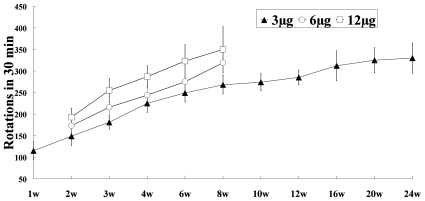
Differential effect of APO on the ST infusion rats. The APO-induced rotations were counted on the 1^st^, 2^nd^, 3^rd^, 4^th^, 6^th^, 8^th^, 10^th^, 12^th^, 16^th^, 20^th^, and 24^th^ weeks following the 3-µg rotenone infusion and on the 2^nd^, 3^rd^, 4^th^, 6^th^, and 8^th^ weeks following the 6-µg and 12-µg rotenone infusions. The control animals did not exhibit rotations. The total number of rotations is expressed as the mean±SD (n = 6).

ST-infused rats demonstrated typical contralateral rotations following APO injections at all time points. The rotational behavior lasted for one hour after the APO administration, and the rotational behavior was dose-dependent in the 3-, 6-, and 12-µg groups. In the 3-µg group, 30 minutes after the APO administration, the rotation number was 115±21, 149±22, 181±16, 225±21, 249±21, and 268±21 at the first, second, third, fourth, sixth, and eighth week, respectively, and the change was time-dependent (Figure 1). The total number of rotations increased gradually until the twenty-fourth week (330±35).

### DA Is Depleted in the Early Stage, but 5-HT Is Decreased in the Late Stage

**Figure 2:**
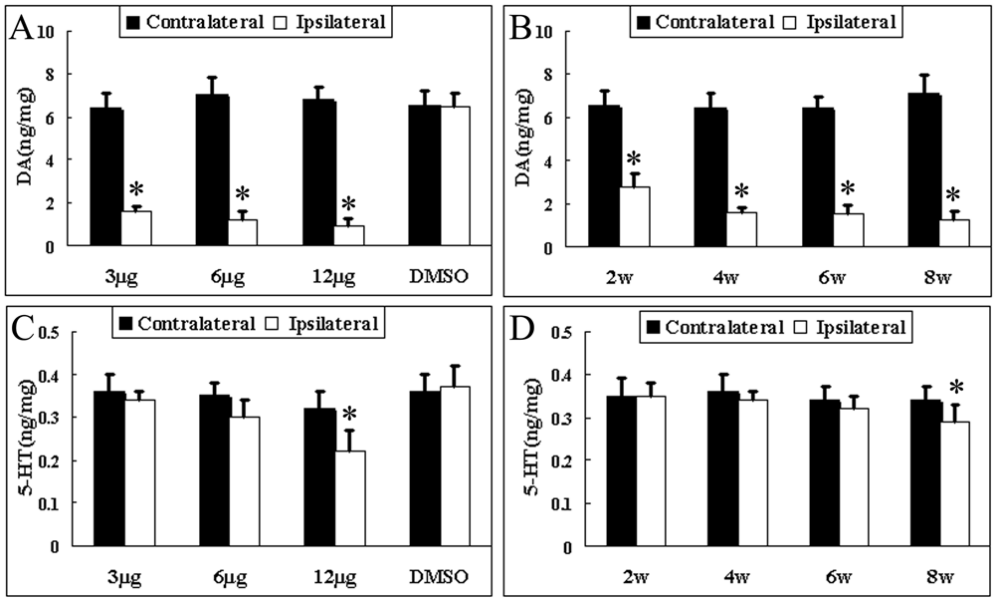
Changes in striatal levels of DA and 5-HT after rotenone infusion. For the dose-response (3-, 6-, 12-µg groups) studies, the animals were sacrificed at the fourth week following the ST infusion of rotenone, and the (A) DA and (C) 5-HT contents were measured. For the time-response studies, the animals were infused with 3 µg of rotenone and sacrificed on the second, fourth, sixth, and eighth week (B and D). The results are expressed in ng/mg (wet tissue), and the data were given as the mean±SD (n = 6). *P<0.05 as compared to contralateral side.

The levels of monoamine neurotransmitters in the left and right striata of rat brains were not significantly different in the control groups. However, the unilateral intracerebral infusion of rotenone at doses of 3, 6, and 12 µg caused significant DA depletion in the ipsilateral striatum in a dose-dependent fashion at the fourth week as compared to the contralateral striatum. For instance, the DA content in the 3-, 6-, and 12-µg groups were reduced to 24.42%, 17.38%, and 13.11% at the fourth week, respectively (Figure 2A). In 3- and 6-µg groups, the 5-HT content did not show any change, but in the 12-µg group, 5-HT was decreased (Figure 2C). For the time-dependence study, there appeared to be a progressive loss of DA in the striatum. At the second, fourth, sixth, and eighth weeks, the DA content was decreased by 57.73%, 75.58%, 76.43%, and 82.68% as compared to the contralateral striata, respectively (Figure 2B). The 5-HT content did not exhibit any significant change during the first six weeks, but it decreased at the eighth week (Figure 2D).

### GSH and SOD Decreased and MDA Generated in Dose- and Time-Dependent Fashions

**Figure 3:**
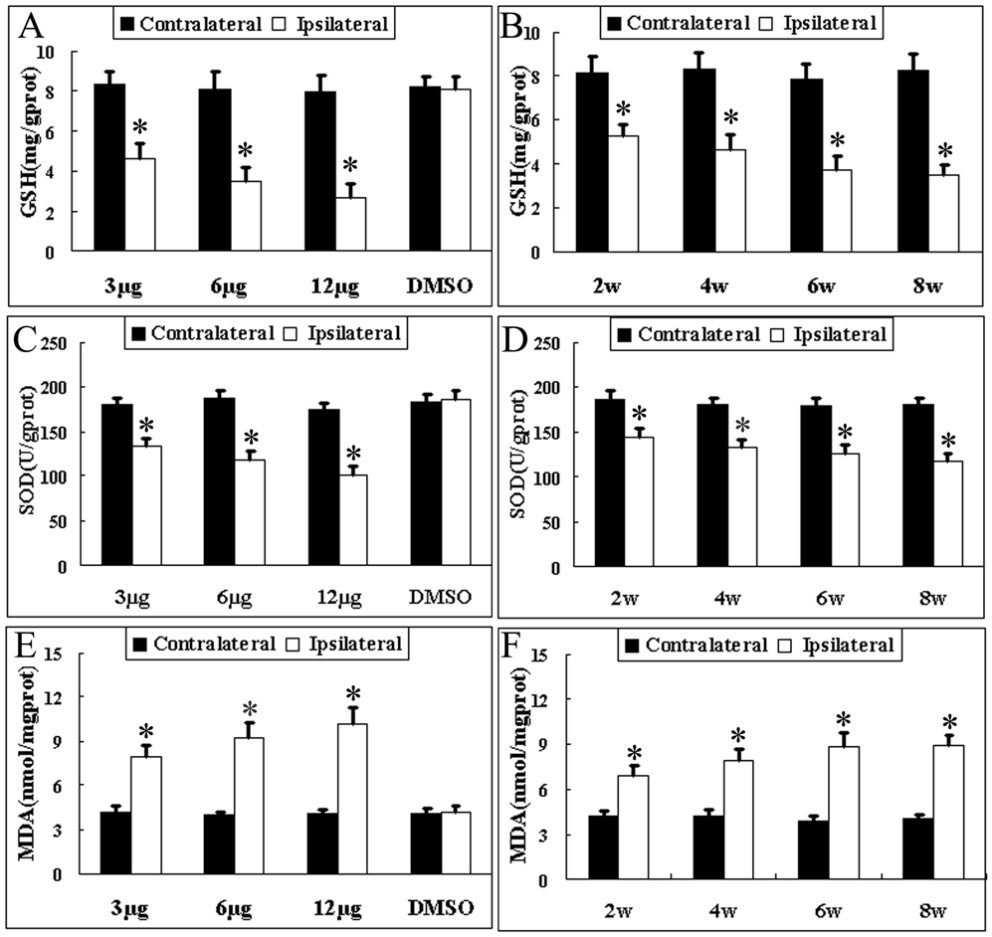
Effects of ST infusion of rotenone on SNc oxidative stress levels. (1) Dose response: the rats were sacrificed on the fourth week following the ST infusion of different doses (3-, 6-, or 12-µg) of rotenone, and the GSH activity (Figure 3A), SOD activity (Figure 3C), and MDA level (E) in the midbrain on the left and right sides were spectrophotometrically measured. (2) Time response: The animals infused with 3-µg rotenone were sacrificed on the second, fourth, sixth, and eighth week for the GSH, SOD, and MDA determination. *P<0.05, as compared to contralateral side. Data were expressed as the means±SD (n = 6).

Four weeks after surgery, the ST infusion of rotenone caused a significant increase in the MDA level and a decrease in GSH and SOD activity in the ipsilateral midbrain as compared to the contralateral side. The vehicle infused midbrain did not experience any change in the MDA level and GSH or SOD activity as compared to the contralateral side. The ST infusion of rotenone at doses of 3, 6, and 12 µg into rats resulted in a significant depletion of GSH and SOD (Figure 3A and 3C) and a generation of MDA in a dose-dependent manner (Figure 3E). The GSH in the ipsilateral midbrain on the fourth week was reduced by 44.03%, 57.54%, and 66.23% in the 3-, 6-, and 12-µg groups, respectively, as compared to the contralateral side; the SOD in the ipsilateral midbrain on the fourth week was decreased by 26.64%, 36.49%, and 42.39%, respectively; and the MDA was increased by 86.55%, 131.53%, and 152.44%, respectively. The GSH activity in the ipsilateral midbrain on the second, fourth, sixth, and eighth weeks after infusion was diminished by 34.88%, 44.03%, 52.49%, and 57.62%, respectively (Figure 3B); the SOD activity was decreased by 22.35%, 26.64%, 29.46%, and 35.13%, respectively, while the MDA levels were increased by 64.93%, 86.55%, 124.28%, and 122.36%, respectively(Figures 3D and 3F), indicating a slow and time-dependent change in these three oxidative stress markers.

### Nigrostriatal TH Immunoreactivity Decreased and Alpha-Synuclein Expression Increased

**Figure 4:**
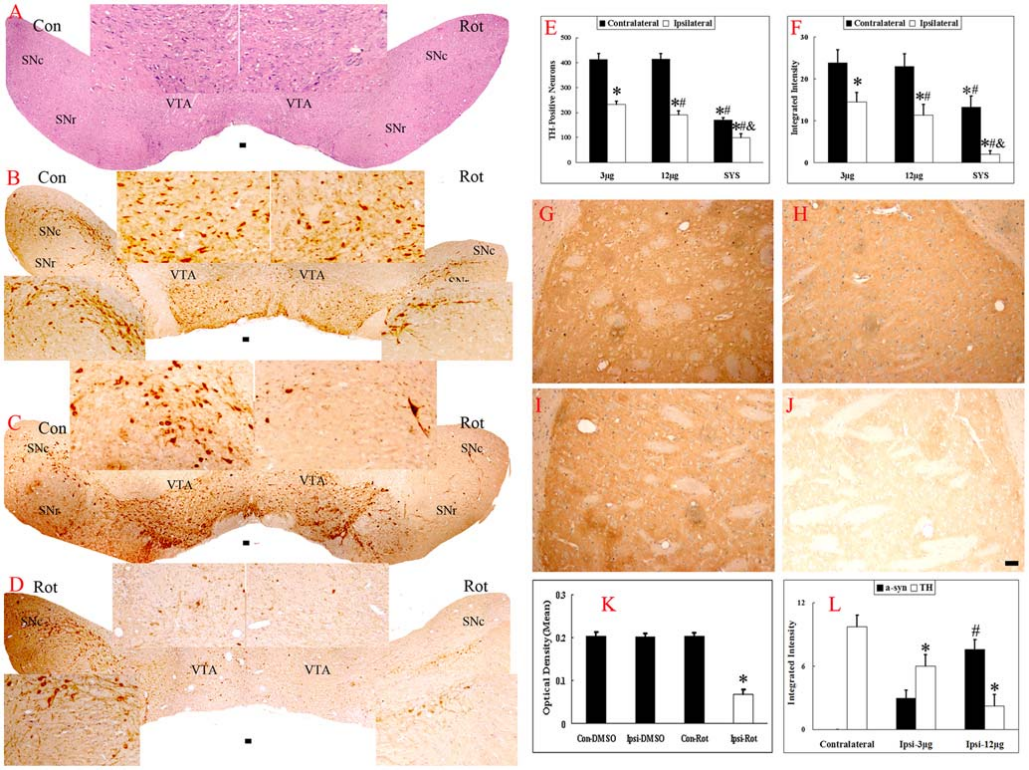
Effect of rotenone on the striatum, VTA, and SNc TH immunoreactivity. Pictures of the TH immunostaining of coronal sections were taken at the level of the striatum from the DMSO- or rotenone-infused rats (3-µg group) four weeks after infusion. Each treatment group had four rats. The left striatum represents the intact side of the brain, and the right striatum represents the lesioned side of the same animal. The TH immunoreactivity on the ipsilateral side of the striatum was decreased by 66.4% (Figure 4I–J; *P<0.05, as compared to the contralateral side). The ST-infused rats (3- and 12-µg groups) and SYS models were sacrificed four weeks after the rotenone infusion, and coronal sections were HE-stained and immunohistochemically stained with TH. HE-staining showed a significant decrease in the number of neurons in the VTA and SNc (A, 3 µg group). The number of TH-positive neurons (E) was decreased by 43.7% (B), 53.6% (C), 59.0%, and 75.8% (D) in the right side of the 3- and 12-µg ST models and in the left and right sides of SYS models, respectively, as compared to the contralateral side of the ST models. The integrated intensity of the VTA and SNc was decreased by 38.1%, 54.5%, 46.4%, and 95.3% in the right side of the 3- and 12-µg ST models and in the left and right sides of the SYS models, respectively (F) (*P<0.05, as compared with the contralateral side; #P<0.05, as compared with the lesioned side of 3-µg group; and P<0.05, as compared with the other side of the SYS group) (Scale bars = 50 µm).

**Figure 5:**
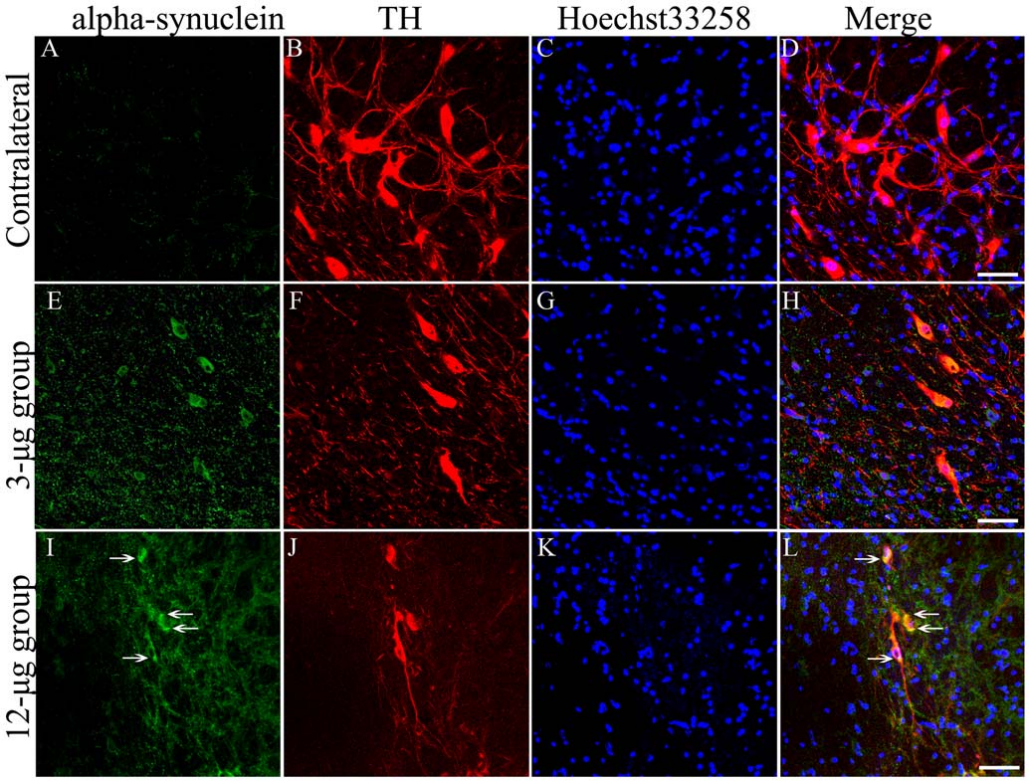
Double immunohistostaining of alpha-synuclein and TH. Samples of the double immunohistostained alpha-synuclein and TH of the lesioned SNc were taken from the rats sacrificed four weeks after surgery (3-µg group: A–H; 12-µg group: I–L). Alpha-synuclein was visualized by FITC-conjugated donkey-anti-mouse IgG, TH was labeled by the Cy3-conjugated goat-anti-rabbit IgG, and the nucleus was stained by the Hoechst 33258. The last graph of the three rows was obtained by the overlapping the first three graphs (Scale bars = 50 µm). Compared with the contralateral side of the ST models (A-D), a dose-dependent increase in alpha-synuclein expression in the lesioned brain and a decrease in the number of TH-positive neurons are shown in E–H (3-µg group) and I–L (12-µg group). The quantitative data were shown as Figure 4L.

The histological examination of the brain tissues from the rats indicated that the vehicle infusion did not change the intensity of the TH immunostaining in the striatum (Figure 4G–H), while the TH staining intensity was significantly decreased (by 66.4%) in rats infused with rotenone (Figure 4I–J). The number of TH-positive neurons was decreased in the ipsilateral VTA and SNc as compared to the contralateral side. The finding was further confirmed by HE staining of VTA and SNc (Figure 4A). The death rates of the TH-positive cells in the VTA and SNc were 28.72% and 58.53%, respectively, as compared to the contralateral side in 3-µg group, while the death rates was 33.47% and 73.73% in the 12-µg group. The double immunohistostaining of alpha-synuclein and TH showed a dose-dependent decrease in the number of TH-positive neurons, an increase in alpha-synuclein expression in the lesioned brain (Figure 5). The quantitative analysis indicated that alpha-synuclein expression in the SNc was up-regulated by 156.1% in 12-µg group as compared to 3-µg group (Figure 5E, I and Figure 4L), while the TH immunoreactivities in the lesioned SNc were decreased by 38.6% and 77.3% as compared to the contralateral SNc (Figure 5B, F, J and Figure 4L).

### Abnormal Mitochondria Associated with DA Neuron Degeneration

**Figure 6:**
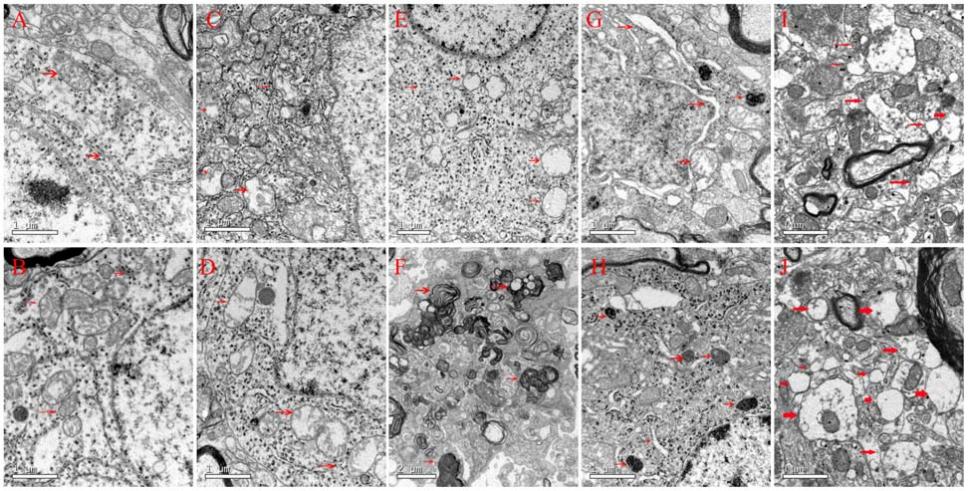
Ultrastructural changes of the SNc. The ultrastructural changes of the SNc were set in order: (1)mitochondrial swelling, (2)mitochondrial crest fracture, (3)mitochondrial vacuolar degeneration, (4)dilated and broken rough endoplasmic reticula, (5)liberation of ribosomes from the rough endoplasmic reticula, (6)lipofuscin deposition, (7)perinuclear space augmentation. A and B: normal mitochondria, rough endoplasmic reticulum. and ribosomes in the contralateral SNc of the 3-µg group animals one week or four weeks after surgery; C: (1), (2), (4) and (5) in lesioned SNc of the 3-µg group animals one week after surgery; D: (1), (2) and (3) in the lesioned SNc of the 3-µg group animals two weeks after surgery; E: (1), (2), (2), (4) and (5) in the lesioned SNc of the 3-µg group animals three weeks after surgery; F: (6) in the lesioned SNc of the 3-µg group animals one week after surgery; G: (1), (2), (3) and (7) in the lesioned SNc of the 3-µg group animals four weeks after surgery; H: the rough endoplasmic reticula and increased lysosomes in glial cells of lesioned SNc of 3-µg group animals four weeks after surgery; I and J: (1), (2) and (3) in both sides of the SNc of the SYS model four weeks after rotenone administration.

The left and right SNc (from −4.5 to −6.2 mm caudal to the bregma) of the SYS group and the 3-µg group were micro-punched and examined under a transmission electron microscope. No conspicuous ultrastructural changes were found in the contralateral SNc (Figure 6A–B), where as mitochondrial swelling, mitochondrial crest fracture, mitochondrial vacuolar degeneration, dilated and broken rough endoplasmic reticula, liberation of ribosomes from rough endoplasmic reticula, lipofuscin deposition, and perinuclear space augmentation were observed in neurons of lesioned SNc (Figure 6C–J). Occasionally, the rough endoplasmic reticula were dilated, and lysosomes were increased in glial cells (Figure 6H).

### Peripheral Organs Alterations in the SYS Model

**Figure 7:**
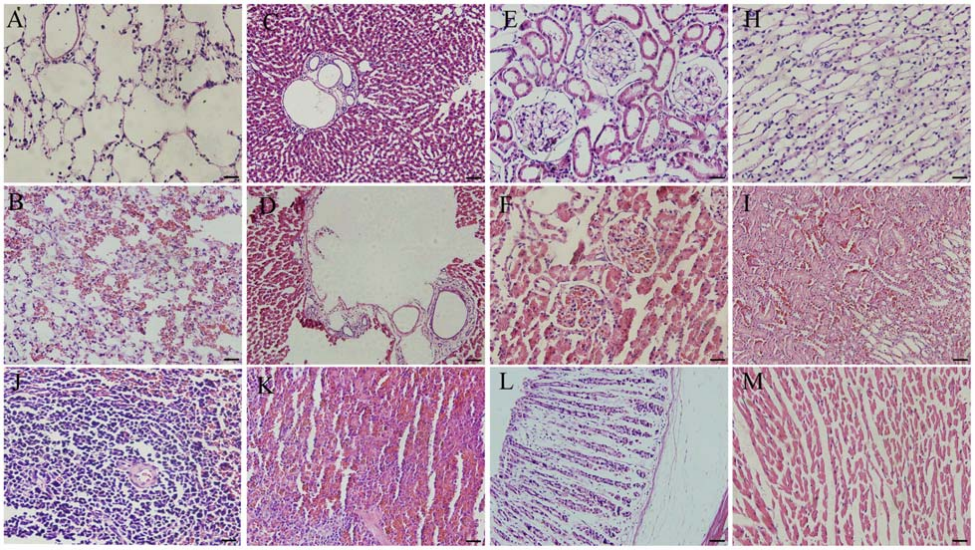
Pathological changes in peripheral organs in Parkinsonian rats. A: The alveolar walls of the ST infusion rats were thin and delicate; B: neutrophilic exudates were detected in the pulmonary alveoli; C: the normal hepatic lobules and central veins; D: the central vein of the hepatic lobule was fractured in SYS rats; E and H: the normal renal glomerulus and renal medulla; F and I: Red blood cells were increased in the renal glomeruli and renal medulla; J: the normal white pulp of the spleen; K: hemorrhage and hemosiderin deposition in the spleen of the SYS rats; L and M: normal heart and stomach of SYS rats (Scale bars = 50 µm).

Among the 12 SYS-infused rats, no animals died spontaneously within four weeks of the systemic rotenone administration (2.0 mg/kg/day). In these SYS rats, significant changes were observed in the liver, kidney, lung, and spleen, while there were no obvious changes in the heart and stomach. For the ST models, no changes were found in the peripheral organs of the 3-, 6-, and 12-µg groups up to the fourth week. In the SYS models, neutrophilic exudates were detected in the lung (Figure 7B), while the alveolar walls of the ST infusion rats were thin and delicate (Figure 7A). In the liver, the central veins of hepatic lobules were fractured in the SYS models (Figure 7D), but they were intact in the ST models (Figure 7C). In the kidney, the number of red blood cells was increased in the renal glomeruli (Figure 7F) and renal medulla (Figure 7I), while few red blood cells were seen in the renal glomeruli of ST animals (Figure 7E and Figure H). In the spleen, hemorrhage and hemosiderin deposition were observed in the SYS infusion rats (Figure 7K), while white pulp was noted in the spleen (Figure 7J). The SYS infusion of rotenone exerted little influence on the heart (Figure 7M) and stomach (Figure 7L) of the SYS infusion rats.

## Discussion

Currently available neurotoxin-based models failed to reproduce the progressive nature and the extensive pathological involvement in PD. Moreover, these models are of limited value due to some significant limitations, such as a rapid loss of DA neurons, a failure to induce typical brain lesions, an absence of alpha-synuclein expression, and the potential of systemic toxicity. Furthermore, most of the current genetic murine models are in the early-stage of PD and fail to demonstrate the characteristic loss of dopaminergic cells observed in PD. In this study, we developed rat PD models by ST and SYS administration of rotenone and made comparisons between these two models. We examined behaviors, brain histology, peripheral toxicity, biogenic amine levels in the striatum, the ultrastructural changes of the SNc, and the oxidative stress levels of the midbrain. In summary, the main features of our current model include: 1) a one-time, low-dose ST infusion of rotenone could generate a characteristic decrease in the nigrostriatal TH immunoreactivity; 2) APO-induced rotations progressed gradually until the 24^th^ week; 3) rotenone depleted DA, but not 5-HT at the beginning in the low-dose groups; 4) the GSH and SOD activities were lowered, and the generation of MDA was increased in the midbrain treated with rotenone; 5) alpha-synuclein expression was increased, and TH-positive neurons were decreased, both in a dose-dependent fashion; 6) the infused SNc developed characteristic ultrastructural changes of abnormal mitochondria; 7) the SYS model can reproduce the typical behaviors of PD but is associated with peripheral toxicity; and 8) after rotenone infusion for four weeks, all the ST model rats and ten of the twelve SYS models showed representative behavioral features of PD. No animals accidentally died during the study. These results demonstrate that acute stereotaxical administration of a small dose of rotenone can induce long-lasting neurochemical and neuropathological PD-like changes in the nigrostriatal system.

### ST Model

Spontaneous contralateral rotation following intracerebral administration of MPP^+^ [39] or ferrous citrate[40] under the same situation has been reported in rats. Recent studies have shown that spontaneous rotation can be induced by intra-nigra infusion of the rotenone, but not by intra-MFB infusion of rotenone. The rotation might be caused by the release of DA into the ipsilateral striatum after rotenone infusion[29]. The spontaneous rotational behavior can also serve as a measure for assessing the success of the animal model. The result of the APO-induced contralateral rotations was different from a previous study in which the infusion of rotenone into the SNc alone caused ipsilateral rotations. The difference might be due to intact VTA neurons in the intra-nigra models. On the other hand, the APO injection at the fourth week following the rotenone infusion failed to produce any significant stereotypical circling behavior in MFB-lesioned rats [29], which makes it difficult to assess the model in the early stage. Moreover, according to previous studies which indicated APO-induced rotations in PD rats should be over 210 in the first 30 minutes [12], [28], [29], [37], we speculated that four weeks after surgery may be the best time point to detect the change of nigrostriatal TH immunoreactivity.

The HPLC analysis of the DA and 5-HT contents suggested that rotenone selectively and specifically acts on the dopaminergic, but not on the serotonergic system; this finding was also seen in an earlier study [28]. Significantly decreased 5-HT contents in the striatum by high-dose rotenone or long-term low-dose rotenone should contribute to extensive pathological involvement in the serotonergic system seen in idiopathic PD[17], which is responsible for the non-motor symptoms including depression [18]. Another important finding about the GSH and SOD activity and MDA level, which is similar to previous results [41], [42], [43], is that specific effects of rotenone on the electron transport chain could cause an increase in oxygen free radicals, thereby resulting in oxidative stress and ultimately leading to severe damage of the DA neurons.

Since the pathological changes in these models included both a loss of the TH-positive neurons and a decrease in the TH expression in the lesioned side, we not only counted the number of TH-positive cells but also measured the integrated intensity of both sides of the VTA and SNc. The finding that the death rate of TH-positive cells in the VTA was substantially lower than that in the SNc is yet another piece of evidence that the DA neurons in the VTA are more resistant to damage and is consistent with previous studies [44], [45]. In SYS models, the most interesting finding was the asymmetric loss of TH-positive neurons in the two sides of the brain, which is very close to the unilateral onset of human idiopathic PD. Moreover, the double-immunohistostaining study suggested that rotenone-induced alpha-synuclein expression was increased in a dose-dependent manner.

Previous animal studies on PD seldom dealt with the ultrastructural changes of the SNc. In this study, the most conspicuous changes in the ST model included mitochondrial swelling and crest fracture, as found in the SYS model. The ultrastructural change in the lysosome system was another important finding. It is acknowledged that the autophagy-lysosome pathway plays a pivotal role in the pathogenesis of many neurodegenerative disorders including PD[46]. Lysosomes remove the damaged mitochondria by means of phagocytosis, forming autophagosomes. The rough endoplasmic reticula were dilated and lysosomes were increased in even glial cells, suggesting that glial cells may be involved in the pathogenesis of PD, which may provide us with a therapeutic alternative for PD[12].

### SYS Model

In 2000, it was first reported that, similar to the action of MPTP, chronic systemic rotenone exposure reproduced features of PD[15]. Since then, research efforts have been directed at rotenone-induced PD, and the results have been encouraging. The rotenone-induced PD animal model reproduces most motor symptoms and the histopathological changes of PD including Lewy bodies [20], [21], [22]. In contrast, the validity of rotenone-induced PD symptoms has been challenged by some researchers [25], [26]. However, our previous research showed that subcutaneously infused rotenone was an effective method to reproduce PD in rats [20]. In SYS models, a progressive loss of body weight (by 2–5 g/d, data not shown) and yellow discoloration of the skin were also observed. These changes might have been caused by difficulty in eating and skin-cleaning associated with behavioral changes. Two rats did not develop the behavioral features after the four-week rotenone SYS infusion, but their body weight decreased gradually (by 2–5 g/d). After treatment with SYS-infused rotenone for another four weeks, they developed behavioral changes. We found that rotenone could accumulate in the subcutaneous adipose tissue because of its lipophilic nature, which might, in part, explain the variation in susceptibility among the animals. When the animals lose body weight, rotenone is released from the fatty tissues and then enters body fluid circulation, thereby causing systemic toxicity, which may be responsible for the high death rate of the SYS models. The significant pathological changes observed in the peripheral organs of SYS models were different from previously reported findings with Lewis rats [26], in which changes in the stomach were predominant. This difference might be due to the fact the Sprague-Dawley rats had stronger survival ability, and different chows were used.

The most commonly used 6-OHDA and MPTP models are generally acute and can produce a rapid loss in terminals and a decrease in TH-positive cells [21]. Moreover, 6-OHDA and MPTP are very difficult to store and must be used immediately after being dissolved. The ST model can definitely reproduce slow and specific nigrostriatal dopaminergic degeneration in association with the formation of Lewy bodies and the progression in APO-induced rotational behavior, which so far has not been found with 6-OHDA models and only reported once with MPTP models [47]. We came to the conclusion that the Parkinsonian animal model produced by ST infusion of rotenone into both the VTA and SNc is effective for the study of the behavioral syndromes, the molecular mechanism of Lewy body formation, and the link between this formation and nigrostriatal dopaminergic neuronal degeneration. It can also be used for the screening of anti-Parkinsonian drugs and the development of novel therapeutic and diagnostic methods. Currently, we are using this model for neuroprotective drug screening and stem cell therapy for PD.

**Table 1:** Comparison of our rotenone ST models with other models.

	Rot ST	MPTP ST[48]	Rot SYS[15], [20], [49]	MPTP SYS[47], [50], [51], [52], [53]
**Animal**	**Rat**	Rat	Rat	Mouse
**Instrument**	**ST**	ST minipump	S.C or Osmotic minipump	S.C or Osmotic minipump
**Inclusion formation**	**Yes**	Only in striatum not SNc	Yes	Only in continuous low-level exposure models[47]
**Nutritional status**	**Good**	Good	Supplementation by gavage	Data not shown
**Peripheral toxicity**	**No**	No	Yes	Theoretically, but data not shown
**Pathogenetic research**	**Yes**	Yes	Yes	Yes
**Pathophysiological research**	**Yes**	Yes	Yes	Yes
**Drug screening**	**Yes**	Yes	Limit	Limit
**Variability**	**Low**	Low-medium	Medium	Low-medium
**Mortality**	**Low**	Low	Medium	Low-medium
**Slow progression**	**Yes**	Yes	Yes, with serious consequence	In some models
**Selective degeneration**	**Yes**	Yes	No	Yes
**Long-term progression**	**Yes**	data not shown	No	In continuous low-level exposure models
**Extensive involvment**	**Yes**	No	Yes, with serious consequence	Yes
**Cost**	**Low**	medium-high	Medium-high	Medium-high

Data on the rotenone ST and SYS administration models are from our study and other studies, while data on the MPTP models are all from reports by others. Animal, animal used for the research; Instrument, special instrument or routes of administration; Nutritional status, the nutritional status of PD animal models and whether supplementation is required; Peripheral toxicity, peripheral organ toxicity caused by the neurotoxin; Application, whether the model is suitable for the study of pathogenesis, pathophysiological research, or drug screening of PD; Slow progression, idiopathic PD-like slow progression; Selective degeneration, selective degeneration of DA neurons; Long-term progression, long-term (more than three months) progression without intervention; Extensive involvement, ability to reproduce extensive pathological involvement observed in PD patients; Cost, costs of neurotoxin drugs, instruments, nutritional supplementation (ST, Stereotaxical Infusion; SYS, Systemic administration; S.C., subcutaneous injection).
